# Free-Ranging Dogs Are Capable of Utilizing Complex Human Pointing Cues

**DOI:** 10.3389/fpsyg.2019.02818

**Published:** 2020-01-17

**Authors:** Debottam Bhattacharjee, Sarab Mandal, Piuli Shit, Mebin George Varghese, Aayushi Vishnoi, Anindita Bhadra

**Affiliations:** ^1^Department of Biological Sciences, Indian Institute of Science Education and Research Kolkata, Kolkata, India; ^2^Department of Environmental Science, University of Calcutta, Kolkata, India; ^3^Department of Zoology, CMS College Kottayam, Kottayam, India; ^4^Department of Biological Sciences, Indian Institute of Science Education and Research Bhopal, Bhopal, India

**Keywords:** interspecific communication, referential gestures, social cognition, distal cues, point following

## Abstract

Dogs are one of the most common species to be found as pets and have been subjects of human curiosity, leading to extensive research on their socialization with humans. One of the dominant themes in dog cognition pertains to their capacity for understanding and responding to human referential gestures. The remarkable sociocognitive skills of pet dogs, while interacting with humans, is quite well established. However, studies regarding the free-ranging subpopulations are greatly lacking. The interactions of these dogs with humans are quite complex and multidimensional. For the first time, we tested 160 adult free-ranging dogs to understand their ability to follow relatively complex human referential gestures using dynamic and momentary distal pointing cues. We found that these dogs are capable of following distal pointing cues from humans to locate hidden food rewards. However, approximately half of the population tested showed a lack of tendency to participate even after successful familiarization with the experimental setup. A closer inspection revealed that anxious behavioral states of the individuals were responsible for such an outcome. Finally, we compared the results using data from an earlier study with dynamic proximal cues. We found that free-ranging dogs follow distal cues more accurately compared to proximal cue. We assume that life experiences with humans probably shape personalities of free-ranging dogs, which in turn influence their responsiveness to human communicative gestures.

## Introduction

Interspecific communication (human–non-human animals), employing directional or referential gestures, has widely been studied in the last two decades. Several non-human animals like chimpanzees and bonobos (Tomasello and Camaioni, 1997; Mulcahy and Call, 2009), orangutans (Zimmermann et al., 2009), horses (Maros et al., 2008; Malavasi and Huber, 2016), seals (Shapiro et al., 2003), elephants (Smet and Byrne, 2013), cats (Miklósi et al., 2005), goats (Kaminski et al., 2005), dogs (Soproni et al., 2001, 2002; Miklósi and Soproni, 2006), and wolves (Udell et al., 2008; Virányi et al., 2008) have been shown to respond to such gestures from humans. Although an initial surge was observed in the investigation of interspecific communication using non-human primates, scientists gradually shifted to testing canids which, in turn, facilitated the development and advancement of comparative research methods. As a result, a great deal of information on interspecific communication and the underlying evolutionary mechanisms were acquired.

Dogs (*Canis lupus familiaris*) are arguably the first species to have been domesticated, at least 10,000–15,000 years ago (Vilà et al., 1997; Savolainen et al., 2002; Frantz et al., 2016). Several studies have found distinct behavioral differences in dogs with regard to their closest living ancestors, the gray wolves (*Canis lupus lupus*) (Miklósi et al., 2003; Gácsi et al., 2005, 2009). Researchers have also highlighted the contribution of other key factors, such as ontogenic experiences and socialization (Wynne et al., 2008; Udell, 2015). Cognitive advancement in the communicative abilities has been observed in domesticated Bengalese finches (Okanoya, 2004). Similarly, complex social skills have evolved in dogs after domestication (Hare and Tomasello, 2005). Pet dogs are remarkably skilled at responding to various human social cues (Hare and Tomasello, 1999, 2005; Soproni et al., 2002; Miklósi and Soproni, 2006). A range of studies has elucidated their ability to comprehend human communicative intents such as pointing gestures (Miklósi and Soproni, 2006; Lakatos et al., 2009; Elgier et al., 2012). Pet dogs, in general, are capable of following human pointing cues, from the simplest (e.g., proximal cues) to the most complex types (e.g., distal cues) (Miklósi and Soproni, 2006; Lakatos et al., 2012). Wolves, on the other hand, have been shown to differ in utilizing human communicative signals, especially the momentary distal cues, because of less socialization and delayed emergence of such behavior (Gácsi et al., 2009). Nonetheless, both genetic predisposition (through domestication) and human socialization (or lifetime experiences) have impacted and shaped the point-following behavior of canids (Lampe et al., 2017). Unfortunately, most studies attempting to understand the abilities of dogs to comprehend human social cues have primarily focused on pet dogs who depend entirely on their owners for survival. Hence, their behavioral outcomes could just be a result of indirect conditioning. While the problem has been dealt with to some extent with studies examining shelter dogs’ response to human pointing cues (Udell et al., 2010; Duranton and Gaunet, 2016), a larger picture can only emerge with quantifying responses of free-ranging dogs, which represent the largest population of dogs in the world (Hughes and Macdonald, 2013).

Free-ranging dogs are found in most of the developing countries and live without direct human supervision (Cafazzo et al., 2010). They are primarily scavengers depending on human leftover food but also display occasional begging from humans (Bhadra and Bhadra, 2014; Sen Majumder et al., 2014). Free-ranging dogs interact with humans regularly and receive both positive (food, social petting, etc.) and negative (beating, harassment, and even poisoning) stimuli. Therefore, these dogs are engaged in situations of conflict with humans in many dimensions (Vanak and Gompper, 2009; Gompper, 2015). Humans have been found to be responsible for causing 63% of early life mortality in free-ranging dogs (Paul et al., 2016). Earlier, we showed that at a population level, free-ranging dogs are aversive while making direct physical contact with unfamiliar humans (Bhattacharjee et al., 2017b). This could simply be a strategy to avoid any unprecedented conflict with humans. Therefore, lifetime experiences may vary and can have a significant impact on the social behavior of dogs. This can also lead to inter-individual differences in dogs in terms of responsiveness to unfamiliar humans. Situation-specific responsiveness toward varying human social cues is evident in free-ranging dogs (Bhattacharjee et al., 2018). They were found to comprehend friendly and varying levels of threatening signals from humans and react accordingly. However, communication using human pointing cues has not been studied extensively. In India, people typically feed free-ranging dogs using two distinct ways – (i) by bending down a bit in the front and (ii) throwing food items away and using pointing cues to help dogs locate the food (generally to avoid direct contact with dogs). Therefore, ecologically relevant studies pertaining to human cues ranging from simple to relatively complex (e.g., proximal cues to distal cues) need rigorous testing. Moreover, such an anthropogenic environment is likely to influence dogs’ understanding of human social signals.

Spatial co-occurrence of local stimuli with the goal helps guide the behavior of animals in proximal or tapping cue conditions, making them easier to follow; however, in a distal cue condition, no cues co-occur with the goal object, requiring spatial learning skills (Morris, 1981). Earlier, we reported free-ranging dogs’ ability to follow dynamic proximal pointing cues in all ontogenic phases – pup, juvenile, and adults (Bhattacharjee et al., 2017a). The study offered two key findings – an effect of ontogeny on the point-following behavior and its plasticity as a function of the reliability of the human experimenter (in adult dogs only). However, we did not quantify the behavioral states or the behavioral expression (e.g., friendly, anxious or fearful, shy, etc.) of the dogs toward the unfamiliar human experimenter, which might also have played an important role in their reactions. Thus, it is essential to examine free-ranging dogs with relatively complex human referential cues focusing on their behavioral states to better understand the nature of interspecific interactions with humans.

In this study, we aim to investigate free-ranging dogs’ ability to understand two specific human pointing gestures – dynamic distal and momentary distal cues (Miklósi and Soproni, 2006). We used behavioral states of dogs as a proxy for their life experience with humans to further understand the responsiveness to such cues. Finally, we compared datasets from an earlier study testing free-ranging dogs with dynamic proximal pointing cues using identical experimental conditions (Bhattacharjee et al., 2017a). The comparative approach was used to draw a more complete picture of these dogs’ point-following behavior. We hypothesize that free-ranging dogs would be able to comprehend distal cues from an unfamiliar human experimenter due to relevance in their day-to-day begging behavior. We also hypothesize that the behavioral states would play a key role in defining the repertoire of free-ranging dogs’ responsiveness to such cues.

## Materials and Methods

### Subjects and Study Sites

We tested a total of 160 adult free-ranging dogs in this study (test: dynamic distal cues = 60, momentary distal cues = 60; control: 40 dogs). All the dogs were randomly located on the streets of Kanchrapara (22°94′41″N, 88°43′35″E), Kalyani (22°58′30″N, 88°26′04″E), and Mohanpur (22°96′05″N, 88°56′74″E), West Bengal, India. Experimenters randomly walked on the streets to locate solitary individuals. All possible urban habitats where dogs can be found such as market places, railway stations, bus stations, and residential areas were sampled. Adult dogs that seemed physically fit (in appearance, without any sign of injuries and wounds) were considered for testing. We took photographs of the dogs, recorded coat color, specific color patches, scar marks, and approximate body size to avoid retesting. We confirmed the sexes of the dogs by observing their genitals (male - 91; female - 69).

### Experimental Procedure

We used a two-way object-choice task, where two experimenters, namely, E1 and E2, were involved and played specific roles. E2 was consistent, while four other people played the role of E1. We used opaque plastic bowls (volume = 500 ml) and cardboard pieces as their covers. Small pieces of raw chicken (roughly 10–12 g) were used as hidden food rewards. Here, we provided adult free-ranging dogs with two types (momentary and dynamic) of distal pointing cues (Miklósi and Soproni, 2006) to locate hidden food rewards. We used a double-blind experimental approach where E2 and the subjects had no prior information regarding the location of the hidden food reward. E2 extended one of his arms only for 1 s toward one of the bowls and provided the momentary cue (Supplementary Movie S1) after which the arm rests at the side or back of the body. In dynamic cue condition (Supplementary Movie S2), the pointing cue was provided throughout the trial. Pointing cues using the left and right arms were counterbalanced. Separate sets of dogs were tested using momentary and dynamic distal cues.

Experimenters walked on randomly selected streets of the study sites to locate solitary free-ranging dogs. Once sighted, E1 lured the individual and carried out an initial familiarization phase. Further experimentation was done only after a successful familiarization phase. The detailed experimental procedure is described below:

#### Familiarization

Free-ranging dogs in India are not habituated to getting food from covered plastic bowls. Thus, this phase was carried out to familiarize them with the bowls used in the experimental setup. E1 carried out this phase for all the individuals without involving E2 (the person providing cues) in the process. E1 showed a raw chicken piece to an individual dog and allowed to sniff it closely, then placed it inside an opaque plastic bowl and covered it with cardboard. E1 placed the covered bowl on the ground at an approximate distance of 1.5 m from the dog and stood 0.5 m behind the bowl. Video recording of the process was done starting from the placement of the bowl and continued for a maximum period of 30 s or until an individual retrieved the food reward, whichever was earlier (Bhattacharjee et al., 2017a). We recorded the videos using a wide-angle Sony HDR PJ410 camera mounted on a tripod. Only the dogs that were successful in retrieving the food were included in the subsequent phases (either test or control phase) of study. We discarded a total of 37 dogs that failed to succeed in the familiarization phase. Selection of subsequent test or control phase was random.

#### Test (Using Dynamic and Momentary Distal Cues)

Following a successful familiarization phase, individuals were tested either with momentary or dynamic distal pointing cues in the test phase. Assignment of the type of cue was performed randomly, and we ensured that no dogs were retested with a different cue.

At first, E1 placed a food reward randomly in one of the bowls, false baited the other one by rubbing the raw chicken piece, and covered both using cardboard pieces. The baiting process was not shown to E2 and the focal dog, thereby maintaining the double-blind experimental setup (also see Bhattacharjee et al., 2017a). Therefore, E2 and the dogs had no prior information on the location of the hidden food reward. Immediately after that, E1 handed over the covered bowls to E2, who placed the bowls on the ground. The bowls were placed (1 m away from each other) in such a way that they remain equidistant from the focal dog. The approximate distance between the midpoint of the two bowls placed and the focal dog was 1.5 m. E2 moved 0.5 m back from the mid-point of the bowls after placing them on the ground. Since the dogs were not on leash, E2 sometimes had to reposition (by moving) himself before providing the cue to maintain the distances. E2 tried to catch the attention of the focal dog by clapping once. As soon as eye contact was established, E2 pointed randomly at one of the bowls (1–2 s for momentary or 30 s for dynamic, randomly decided). If the focal dog looked away or turned away during pointing, E2 clapped again to attract its attention. Since distal cues were used, the distance between the tip of the pointing finger and the covered bowl was roughly 0.5 m. E2 gazed at the focal dog throughout the trial for both the types of cues. Approach was defined when the dog moved toward any of the bowls (irrespective of the pointing signal) and uncovered it to inspect. Inspecting a bowl within 30 s ended a trial. The other bowl was immediately removed by E2 to avoid further inspection by the dog. If the dog found food reward upon uncovering a bowl, it was allowed to obtain it. E2 revealed the contents of both the bowls to the dog after an approach within 30 s or after completion of the trial, whichever was earlier. However, E2 never allowed a dog to eat the food reward if the dog chose a false-baited bowl. We carried out three consecutive trials with 5- to 10-s intervals in between. E2, sometimes changed his starting position of a trial to maintain the abovementioned distances as the dogs were not on leash. We tested separate sets of 60 dogs with the two types of pointing cues.

#### Control

The control condition was carried out with a different set of individuals (individuals not used for test condition) immediately after the familiarization phase. Here, E2 did not provide any pointing cue, stood in a neutral posture, and made eye contact with the focal dog. The procedure was otherwise the same as explained in the test condition. Control trials were run to rule out further possibilities of olfactory cues and the effect of motion or orientation response hypothesis (Appelle, 1972). The control condition consisted of only a single trial without any repetitions as the reliability of dogs on E2 could only be calculated using test trials. We tested 40 dogs in the control condition.

### Data Analysis

Videos were coded by a single coder, and a naive person also coded some of the videos (22%) to check for coder reliability. We coded the following parameters from the videos – approach to experimental setup, point following, latency of approach to the experimental setup, behavioral states of the individuals, frequency of gaze alternations between the bowls and E2, and the duration of gazing at E2 using only trial 1 data. This step enabled us to remove a bias of learning of the dogs and its potential impact on the later trials. In addition, single-trial-based controls allowed us to do our comparisons with trial 1 data of test conditions more consistently. However, we used data from all three trials to calculate the reliability of E2 on dogs (see later). All the parameters used are described below:

#### Approach

Approach was defined when a focal dog removed the cover of any of the bowls by moving toward it from his/her initial location. A focal dog could approach a bowl with or without following the pointing cue. When a focal dog stayed back in his/her initial position or left the place without inspecting (uncovering) a bowl, it was considered as no approach. Approach was coded as a binary variable.

#### Ability to Approach the Pointed Bowls

Only dogs that approached the experimental setup were considered for analyzing. Point following was defined by the approach of a focal dog toward the pointed bowl. Point-following behavior was coded as a binary variable.

#### Latency of Approach

It was defined as the time elapsed between the moment when the experimenter extended his arm (pointing cue) and a focal dog removed the cover of any of the bowls. Thus, individuals that did not approach the experimental setup had no latencies by default.

#### Frequency of Gaze Alternation

Gaze alternation has been considered as an intentional and referential communicative act in dogs (Merola et al., 2012; Marshall-Pescini et al., 2013). In this study, the frequency of alternation of gaze between the bowls and E2 was counted. We used a three-way gaze alternation method for coding. Therefore, an event of gaze alternation was counted when a focal dog looked at E2 and the bowls or vice versa within 3 s. We did not consider an event as gaze alternation when a focal dog looked away either from the bowls or E2 within the 3-s duration.

#### Duration of Gazing

Gazing is found to be a critical behavior in communication, which can provide valuable context-specific information on animal intentions (Miklósi et al., 2000; Maglieri et al., 2019). Gazing at the upper body (above the waist) of E2 has been assessed. Emphasis was given on the direction of the focal dog’s nose. Eye contact between the focal dog and E2 was not necessary while calculating the duration of gazing. It was cumulative in nature, and hence, total duration was measured.

#### Behavioral States

Dogs were grouped under the following behavioral states:

• *Affiliative*: Proximity-seeking, fast or rapid tail wagging with the tail perpendicular to or below the body plane, ears pointed upward, maintaining eye contact with E2;
• *Anxious*: Ducking posture with tail between hind legs, excessive panting, lip-licking, corners of the mouth retracted down and back;
• *Neutral*: Resting without gazing at E2, lying down, or general disinterest. Approaching E2 without displaying affiliative or anxious responses were also considered within the neutral behavioral state.

#### Reliability

We hypothesize that a dog would rely more on human cues when he/she gets rewarded in a preceding trial by following a pointing cue; similarly, the reliability or the level of trust would reduce if the dog did not receive food after following a human pointing cue. It was measured using the method described by Bhattacharjee et al. (2017a). We used the following parameters to calculate the reliability of E2 – “positive reinforcement” (PR) and “lack of reinforcement” (LR). PR was considered when a dog followed human pointing cue and obtained a reward. LR, on the other hand, depicted the situation when a dog followed a human pointing cue but did not obtain a reward.

We measured the proportion of individuals that followed pointing in a consecutive trial after PR and those that did not follow pointing after LR as measures of behavioral adjustments of dogs. Here, we used data from all three trials of the test conditions in two sets (set 1 – trials 1 and 2; set 2 – trials 2 and 3).

A second person, naive to the purpose of the study, coded 22% of the trials to check reliability. It was perfect for point-following behavior and behavioral states (Cohen’s kappa = 1.00), and almost perfect for latency (weighted Cohen’s kappa = 0.90), frequency of gaze alternations (Cohen’s kappa = 0.94), and gazing duration (weighted Cohen’s kappa = 0.89). Shapiro–Wilk tests were run to check for normality of the data. We found them not normally distributed and performed non-parametric tests throughout. We used the goodness-of-fit chi-square tests to analyze the parameters of approach, point following, behavioral states, and reliability. Latency, frequency of gaze alternation and duration of gazing were analyzed using Kruskal–Wallis tests. *Post hoc* Mann–Whitney *U* tests were carried out using Bonferroni correction. We used a generalized linear model (GLM) analysis using a binomial distribution to investigate the effects of types of pointing cues, behavioral states, and sexes of the individuals on the approach response. We considered approach as the response variable, and types of cues, behavioral states, and sexes as predictors (fixed effects). Akaike information criterion values were considered for selecting the best-fitting model. GLM analysis was performed using “lme4” package of R (version 3.0.2). All other analyses were carried out using StatistiXL (version 1.11.0.0).

## Results

### Approach

50% (30 out of 60), 48% (29 out of 60), and 50% (20 out of 40) of the individuals approached in the dynamic distal cue (test), momentary distal cue (test), and control conditions, respectively. There was no significant difference in the approach responses (goodness-of-fit χ^2^ test: χ^2^ = 0.041, *N* = 160, *df* = 2, *p* = 0.97) between the three conditions.

### Ability to Approach the Pointed Bowl

**FIGURE 1:**
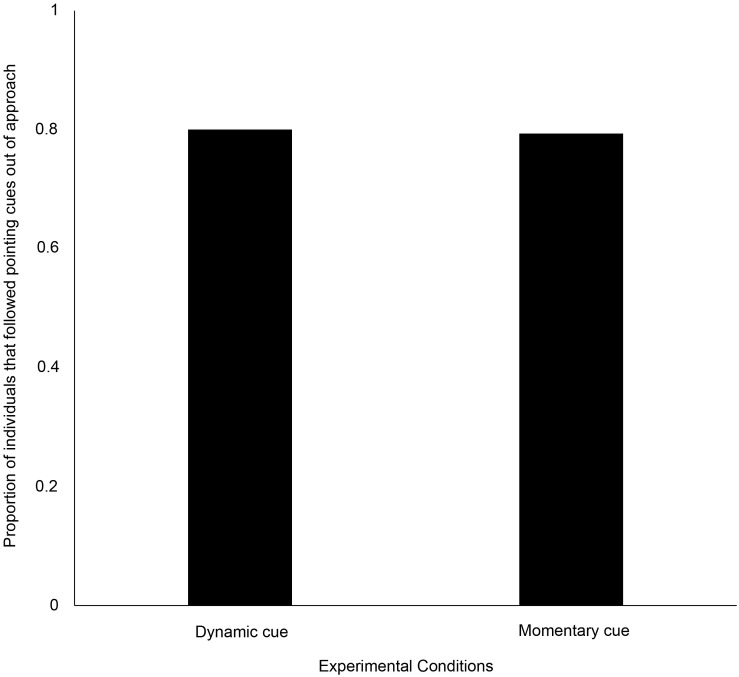
Bar graph showing the proportion of individuals that followed the dynamic and momentary pointing cues.

Out of the individuals that approached, 80% (24 out of 30) and 79% (23 out of 29) of them approached the pointed bowl with dynamic and momentary distal cues, respectively. There was no significant difference between dogs’ point-following behavior using the above two cues (goodness-of-fit χ^2^ test: χ^2^ = 0.000, *N* = 59, *df* = 1, *p* = 1, Figure 1). A significantly higher proportion of individuals followed the two cues, as compared to the proportions who did not (dynamic cue – goodness-of-fit χ^2^ test: χ^2^ = 10.800, *N* = 30, *df* = 1, *p* = 0.001; momentary cue – goodness-of-fit χ^2^ test: χ^2^ = 9.966, *N* = 29, *df* = 1, *p* = 0.002).

Of the dogs that approached (20 dogs) in the control condition, 14 went to the false-baited bowl and 6 to the baited bowl. We did not find the difference to be significant (goodness-of-fit χ^2^ test: χ^2^ = 3.200, *df* = 1, *p* = 0.07). However, when we compared the number of dogs that followed pointing cues and obtained food rewards in the two types of test cues (pooled data), it differed from the number of dogs that obtained food in the control condition (goodness-of-fit χ^2^ test: χ^2^ = 6.857, *df* = 1, *p* = 0.009).

### Latency

Latencies of the individuals that approached did not vary between the test (dynamic and momentary cues) and control conditions (Kruskal–Wallis test: χ^2^ = 3.559, *N* = 79, *df* = 2, *p* = 0.169). In addition, there was no difference in latencies between individuals that followed the dynamic and momentary distal cues (Mann–Whitney *U* test: *U* = 321.000, *N* = 47, *df*_1_ = 24, *df*_2_ = 23, *p* = 0.347).

### Frequency of Gaze Alternation

**FIGURE 2:**
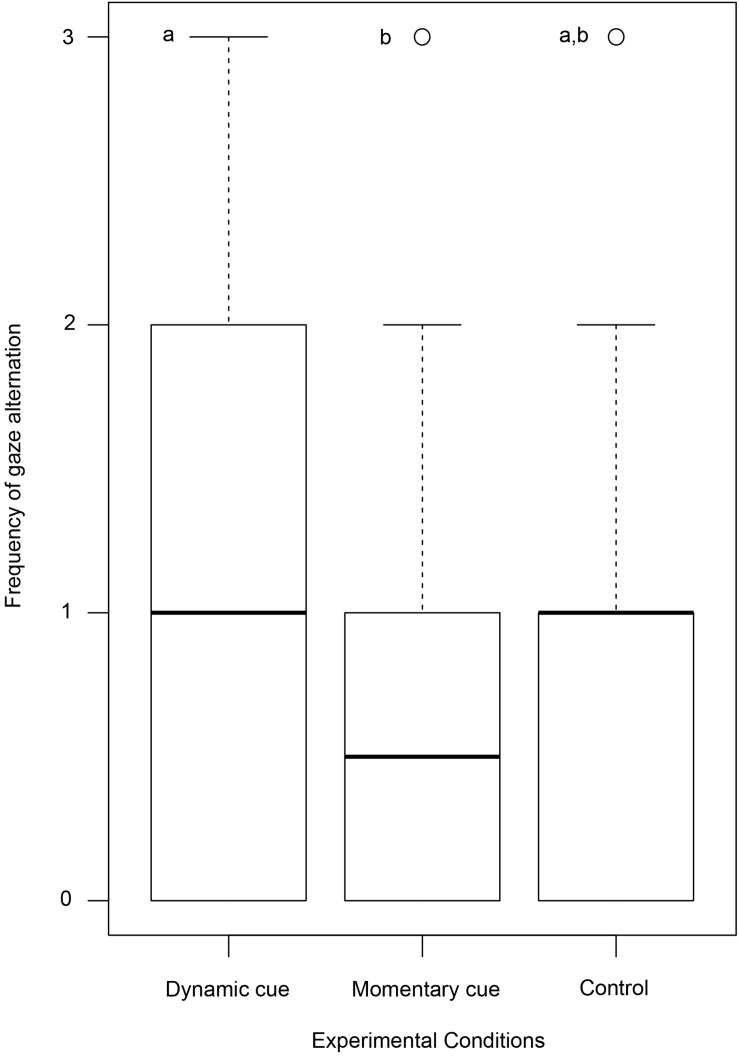
Box and Whisker plot showing the frequency of gaze alternation by dogs. Boxes represent the interquartile range, horizontal bars within boxes indicate median values, and whiskers represent the upper range of the data. Different letters indicate significant differences between the experimental conditions.

We found a difference in the frequency of gaze alternations between individuals in the test (dynamic and momentary) and control conditions (Kruskal–Wallis test: χ^2^ = 11.354, *N* = 160, *df* = 2, *p* = 0.003, Figure 2). *Post hoc* pairwise comparisons with Bonferroni correction revealed a significantly lower frequency of gaze alternations in the momentary cue condition compared to dynamic cue one (Mann–Whitney *U* test: *U* = 2,395.000, *N* = 120, *df*_1_ = 60, *df*_2_ = 60, *p* = 0.002). There was no variation between momentary cue–control condition (Mann–Whitney *U* test: *U* = 1,323.000, *N* = 100, *df*_1_ = 60, *df*_2_ = 40, *p* = 0.390) and dynamic cue–control conditions (Mann–Whitney *U* test: *U* = 1,466.000, *N* = 100, *df*_1_ = 60, *df*_2_ = 40, *p* = 0.06). However, note that the *p* value was just above the significance level (0.05) between the comparison of dynamic cue–control conditions.

### Duration of Gazing

Individuals showed comparable durations of gazing behavior between the test and control conditions (Kruskal–Wallis test: χ^2^ = 0.538, *N* = 160, *df* = 2, *p* = 0.764).

### Behavioral States

In the dynamic distal cue condition, 35, 23, and 47% of the dogs showed affiliative, neutral, and anxious behavioral states (goodness-of-fit χ^2^ test: χ^2^ = 3.100, *N* = 60, *df* = 2, *p* = 0.212), whereas the percentages were 38, 32, and 30%, respectively, for the momentary distal cue condition (goodness-of-fit χ^2^ test: χ^2^ = 7.000, *N* = 60, *df* = 2, *p* = 0.705). We found 17.5, 27.5, and 55% of the dogs to be affiliative, neutral, and anxious in the control conditions (goodness-of-fit χ^2^ test: χ^2^ = 9.050, *N* = 40, *df* = 2, *p* = 0.01). Overall, behavioral states were comparable within the test conditions. Dogs showed higher anxious behavioral states compared to affiliative behaviors in the control condition (goodness-of-fit χ^2^ test: χ^2^ = 7.759, *N* = 40, *df* = 1, *p* = 0.005). Other behavioral states were comparable (neutral–anxious – goodness-of-fit χ^2^ test: χ^2^ = 0.667, *N* = 40, *df* = 1, *p* = 0.05; affiliative–neutral – goodness-of-fit χ^2^ test: χ^2^ = 0.889, *N* = 40, *df* = 1, *p* = 0.34). We further emphasized the anxious behavioral responses and compared test and control dogs. We found that dogs in the control condition were significantly more anxious than in the test conditions pooled (goodness-of-fit χ^2^ test: χ^2^ = 3.967, *N* = 160, *df* = 1, *p* = 0.04).

We emphasized on the test conditions further, pooled the data, and found a significant effect of behavioral states on the approach responses. Approximately 23, 16, and 61% of the individuals that did not approach showed affiliative, neutral, and anxious behavioral states, respectively, with the response levels being significantly different (goodness-of-fit χ^2^ test: χ^2^ = 41.333, *N* = 81, *df* = 2, *p* < 0.001). Fearful or anxious individuals showed higher “no approach” compared to the affiliative (goodness-of-fit χ^2^ test: χ^2^ = 21.314, *df* = 1, *p* < 0.001) and neutral (goodness-of-fit χ^2^ test: χ^2^ = 32.008, *df* = 1, *p* < 0.001) ones. Affiliative and neutral responses were comparable (goodness-of-fit χ^2^ test: χ^2^ = 0.973, *df* = 1, *p* = 0.323).

In addition, out of the 25 individuals that displayed affiliative state, 22 of them (88%) followed pointing cues. Similarly, out of 20 dogs that displayed neutral behavioral state, 16 (80%) individuals followed pointing cues. Finally, out of the 14 dogs that showed anxious behavior, 9 (64%) of them followed pointing cues. We found the responses to be comparable (goodness-of-fit χ^2^ test: χ^2^ = 3.117, *N* = 59, *df* = 2, *p* = 0.21).

### Effect of Sex, Behavioral States, and Type of Pointing Cues on the Approach Response

GLM analysis revealed only a significant effect of anxious behavioral state on the approach response (Table 1). “No approach” was strongly predicted by anxious behavioral states of individuals. We found no effect of sex (GLM: *p* = 0.64) and types of pointing cues.

**TABLE 1:** Generalized linear model (GLM) showing the effects of sex, behavioral states, and types of pointing cues on the approach response (binomial distribution).

	**Estimate**	**Standard error**	***z* value**	**Pr(>| *z*|)**
Coefficients				
Intercept	0.8101	0.5163	1.569	0.117
Sex male	0.1630	0.3567	0.457	0.648
Anxious behavioral state	–1.7787	0.4286	–4.150	3.33e−05^∗∗∗^
Neutral behavioral state	0.2967	0.4466	0.664	0.506
Dynamic distal cue	–0.2409	0.4674	–0.515	0.606
Momentary distal cue	–0.5891	0.4743	–1.242	0.214

*The analysis revealed only a significant effect of anxious behavioral state on the approach response. “No approach” was strongly predicted by anxious behavioral states of individuals. ^∗∗∗^*p* < 0.001.*

### Reliability

We found that individuals adjusted their point-following behavior based on the reliability of E2. However, the effect was only restricted to PR (goodness-of-fit χ^2^ test: χ^2^ = 16.030, *N* = 33, *df* = 1, *p* < 0.001). This was suggestive of dogs’ tendency to follow human pointing cues in a trial significantly more if the individuals followed cues and rewarded in a preceding trial. No effect of the LR was found (goodness-of-fit χ^2^ test: χ^2^ = 2.333, *N* = 21, *df* = 1, *p* = 0.127), suggesting the inability of dogs to adjust their point following behavior when received “misleading cues” (i.e., pointing toward empty bowl).

### Comparison Between Dynamic Distal, Momentary Distal, and Dynamic Proximal Cues

**FIGURE 3:**
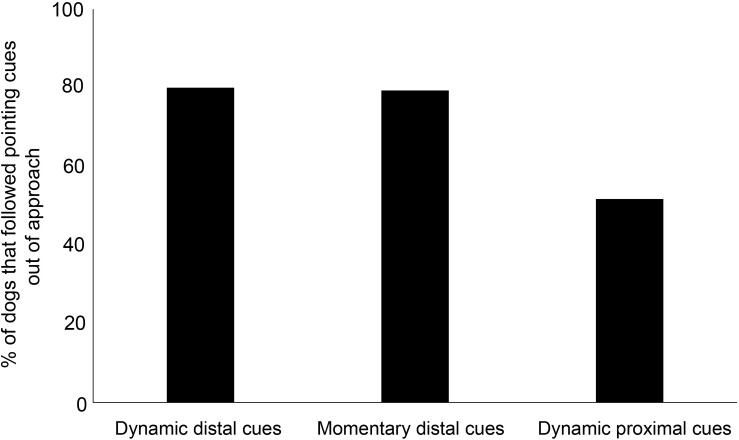
Bar graph showing the percentage of adult dogs that followed the dynamic and momentary distal and dynamic proximal pointing cues.

We compared the proportion of individuals that followed pointing in dynamic proximal, dynamic distal, and momentary distal cue conditions. The comparative analysis revealed a significant difference of the proportion of individuals following pointing cues in the dynamic proximal, dynamic distal, and momentary distal cue conditions (goodness-of-fit χ^2^ test: χ^2^ = 7.2933, *df* = 2, *p* = 0.026, Figure 3). Dogs followed dynamic momentary cues significantly higher compared to dynamic proximal cues (goodness-of-fit χ^2^ test: χ^2^ = 4.075, *df* = 1, *p* = 0.04). However, the responses for dynamic proximal and momentary distal cues were marginally insignificant (goodness-of-fit χ^2^ test: χ^2^ = 3.739, *df* = 1, *p* = 0.05).

## Discussion

Our study showed that free-ranging dogs are capable of following complex pointing cues from humans. Dogs that approached the experimental setup followed both the pointing cues at significantly higher rates, suggesting their ability to rely on complex human referential gestures. Only half of the tested population approached the experimenter, which could be indicative of free-ranging dogs’ population-level perception of humans. Anxious dogs were mostly reluctant to approach the unfamiliar human experimenter even after succeeding in the familiarization phase, whereas their neutral and affiliative counterparts showed significantly higher approach. The varying responses in approach can be explained by dogs’ lifetime experience (with unfamiliar humans), differences in motivation to participate, and the inability to use the referential property of human pointing. We nullify the second possibility as dogs that did not approach in the test or control trials participated in the familiarization phase earlier, so a lack of motivation cannot be the reason for this response. In addition, free-ranging dogs are scavengers and are generally expected not to be well fed (personal observation). We also discard the last possibility as our findings clearly suggest that these dogs can indeed follow distal pointing cues. It is also important to note that the approach rate was also 50% in the control condition where no cue was provided. Thus, the most plausible explanation would be that the behavioral states of the individuals modulated their responsiveness. The initial approach in the familiarization phase was possibly observed because the dogs were allowed to sniff the food reward and watch the baiting process, thus being certain of the reward before approaching. However, in the later phases (either test or control), the uncertainty of getting a reward along with a longer duration of encountering an unfamiliar human could have deterred the anxious individuals from approaching the setup.

The comparative approach (using dynamic proximal, dynamic distal, and momentary distal cues) highlighted a lower tendency of dogs to follow dynamic proximal cues. Since the experimental design was comparable for all the cues, we believe that the type of cue itself (dynamic proximal cue) had affected dogs’ responses. In “Introduction” section, we have mentioned two different ways by which free-ranging dogs in India typically obtain food from humans. While this has not been extensively tested, it is likely that dogs are more accustomed to humans throwing a piece of food away from themselves as a response to begging, or to a human putting/dropping food on the ground and moving away. The complex pointing gestures used in the current experiments simulate these situations quite closely. However, though the proximal pointing cue is considered to be a simpler cue to follow from a completely anthropomorphic perspective to an untrained dog, this might be a more “difficult” situation, with an unfamiliar human constantly pointing at the container, and thereby being in very close proximity to the food source. Adult free-ranging dogs are known to maintain a certain distance from unfamiliar humans and avoid making contact with them (Bhattacharjee et al., 2017b, 2018). It is thus likely that a reduced perception of threat elicited a higher response by the dogs to the distal cues, although the proximal cue is likely to be more definitive and less ambiguous as a signal.

Gaze alternation has been suggested as an intentional and referential act in dog–human communication (Virányi et al., 2006; Marshall-Pescini et al., 2009; Gaunet and Deputte, 2011). Free-ranging dogs displayed comparatively lower frequency of gaze alternations in the distal momentary cue condition as compared to the distal dynamic one. This can be explained by the involvement of higher movements in the dynamic distal cue conditions, which might have influenced the dogs to alter their gaze accordingly. Interestingly, free-ranging dogs have recently been found to discriminate between active and inactive human attentional states and at the same time differ in responses compared to pet and shelter dogs (Brubaker et al., 2019). It seems that the dogs in the streets have been well adapted to using human-directed gazing and gaze alternations. Pet dogs have been found to be deceived by incorrect or wrong cues (Szetei et al., 2003; Prato-Previde et al., 2008; Marshall-Pescini et al., 2011), but they also have some understanding of human reliability (Szetei et al., 2003; Scheider et al., 2013; Takaoka et al., 2015). In an earlier study, we reported free-ranging dogs’ ability to adjust their point-following behavior based on the reliability of the human experimenter (Bhattacharjee et al., 2017a). Here, we found similar outcomes for the complex cues, in spite of the cues being more subtle than the proximal one, further supporting and strengthening the earlier claim.

This study confirms our earlier reports on free-ranging dogs’ ability to follow human gestures, in spite of having no training. They show a high degree of behavioral plasticity in their response to unknown humans, and this suggests a critical role of learning during ontogeny in the dogs. It is possible that largely negative experiences with humans during their early development make dogs more wary of humans, while those dogs that experience positive human interactions early in life are more friendly and approachable. We suggest that humans play a role, albeit inadvertently, in shaping the personalities of free-ranging dogs. This conjecture is supported by a recent study in which we observed that dogs respond differently to unfamiliar humans calling out to them in areas that differ in human flux – dogs in areas of intermediate human flux are more friendly and approachable than those in low and high human flux zones (Bhattacharjee et al., 2019, under review). In India, dog–human conflict is a major problem in many urban areas, and very little is understood about how humans influence the behavior of dogs on streets. The free-ranging dogs have existed on Indian streets for centuries and are excellent urban adaptors (Debroy, 2008). Understanding the dynamics of the dog–human relationship in the urban environment can help in better management of conflict as well as provide insights into urban adaptation in general.

## Data Availability Statement

The datasets generated for this study are available on request to the corresponding author.

## Ethics Statement

The animal study was reviewed and approved by IISER Kolkata Animal Ethics Committee (approval no. 1385/ac/10/CPCSEA). Written informed consent was obtained from the individual(s) for the publication of any potentially identifiable images or data included in this article.

## Author Contributions

DB and AB designed and conceived the study. SM, PS, MV, and AV carried out the field experiments. SM played the role of consistent experimenter (E2). DB analyzed the data and wrote the first draft of the manuscript. AB edited the manuscript and supervised the entire work.

## Conflict of Interest

The authors declare that the research was conducted in the absence of any commercial or financial relationships that could be construed as a potential conflict of interest.
