# Supplementary material for: Free-Ranging Dogs Are Capable of Utilizing Complex Human Pointing Cues
Source: Front Psychol. 2020 Jan 17;10:2818. doi: 10.3389/fpsyg.2019.02818 (PMC6978287; doi:10.3389/fpsyg.2019.02818)
Supplement: Supplementary file 1 [file Data_Sheet_1.pdf]

Supplementary Material

Movie S1 Video showing a human experimenter providing a momentary distal pointing cue to a free-ranging dog. The dog is exhibiting an affiliative response towards the experimenter.

Movie S2 Video showing a human experimenter providing a dynamic distal pointing cue to a free-ranging dog. The dog is exhibiting an anxious response towards the experimenter.
